# HER2-targeted therapy combined with multidisciplinary management in advanced gallbladder cancer: a case report with 90-month survival

**DOI:** 10.3389/fonc.2025.1669818

**Published:** 2025-11-10

**Authors:** Yulin Wang, Guangwei Tian, Yu Cheng, Shuang Wu, Yanfang Wei, Shize Fan, Jinglei Qu, Jin Wang

**Affiliations:** 1 Department of Medical Oncology, the First Hospital of China Medical University, Shenyang, China; 2 Provincial Key Laboratory of Anticancer Drugs and Biotherapy of Liaoning Province, the First Hospital of China Medical University, Shenyang, China; 3 Clinical Cancer Research Center of Shenyang, the First Hospital of China Medical University, Shenyang, China; 4 Department of Radiation Oncology, The First Hospital of China Medical University, Shenyang, China

**Keywords:** gallbladder cancer, anti-HER2 targeted therapy, multidisciplinary management, local therapies, CA199

## Abstract

Biliary tract malignancies are highly lethal, with a 5-year overall survival (OS) rate of less than 10%. This report describes a patient with HER2-positive advanced gallbladder cancer who achieved an OS of 90 months through multidisciplinary interventions and anti-HER2 targeted therapy. The patient achieved a prolonged period of disease stability, who was treated with various anti-HER2 targeted therapy, such as trastuzumab combined with pyrotinib and HER2-targeted antibody-drug conjugate (ADC). The patient received multiple local treatments (e.g., surgery, cryoablation, CyberKnife, and particle implantation) to further control the disease progression. Dynamic CA199 levels paralleled the treatment efficacy. This report underscores the significance of molecular profiling-guided personalized therapy and integrated multidisciplinary management in the treatment of biliary tract cancers.

## 1 Introduction

Biliary tract cancers (BTCs), including gallbladder carcinoma (GBC), represent aggressive malignancies with a dismal prognosis, as evidenced by a five-year survival rate below 10% (1). Systemic chemotherapy options remain limited, with gemcitabine-based regimens offering only modest survival improvements, consistent with Asian patients (2–4).Recent advances in molecular profiling have revealed actionable targets such as human epidermal growth factor receptor 2 (HER2). HER2 alterations, including amplification, overexpression and other rare mutations, are present in approximately 15% of BTCs encompassing GBC (4). While anti-HER2 therapies have revolutionized the treatment for breast and gastric cancers, their role in BTCs (3) remains under investigation, with objective response rates (ORRs) varying widely (23%-47%) (5). Results from clinical trials such as MyPathway (6), HERB (7), HERIZON-BTC-01 (8), KCSG-HB19-14 (9), and DESTINY-PanTumor02 (10) indicate that HER2-targeted therapy for BTCs shows a median progression-free survival (PFS) of 4.0-5.1 months and a median OS of 7.0-15.5 months. By contrast, the patient in this case achieved a PFS of 18 months with dual HER2-targeted therapy (trastuzumab combined with pyrotinib) and ultimately reached an OS of 90 months through sequential anti-HER2 treatment and multidisciplinary interventions, significantly surpassing the median PFS and OS observed in the aforementioned clinical trials. This outcome underscores the substantial potential of precision medicine in the treatment of cholangiocarcinoma. Two phase III trials, TOPAZ-1 and KEYNOTE-966, have evaluated the efficacy of combining chemotherapy with immunotherapy agents (durvalumab and pembrolizumab, respectively) in intrahepatic or extrahepatic cholangiocarcinoma, including GBCs (11, 12).

## 2 Case description

A 59-year-old male patient presented with elevated CA199 levels (1200 U/mL). Computed tomography (CT) imaging of the abdomen revealed a reduction in the liver density, and further improvement of positron emission tomography–computed tomography (PET-CT) imaging suggested metabolically active lesions within hepatic segments.

**Figure 1:**
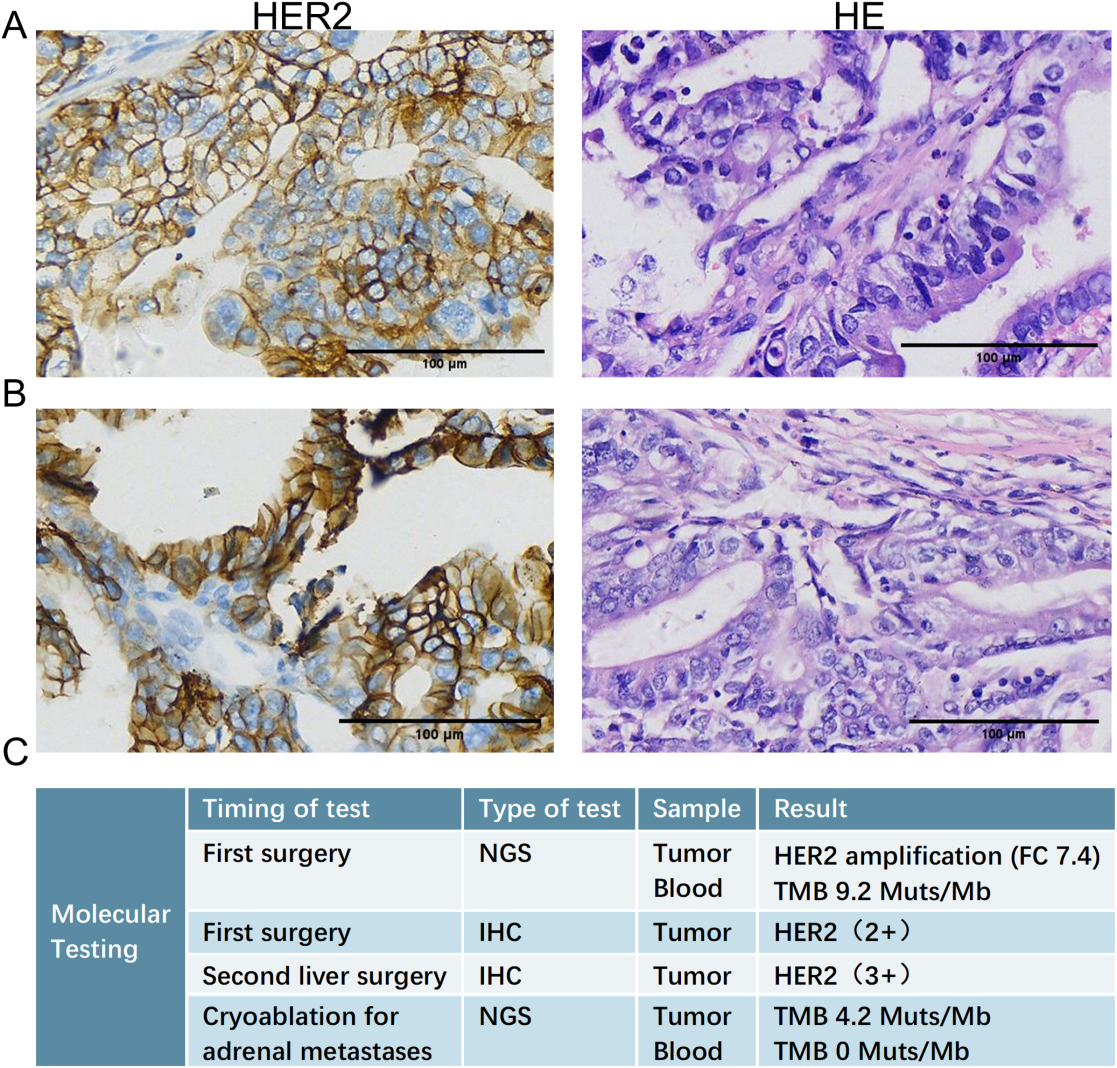
Histopathological examination of liver metastases. **(A)** HE (left) and HER2 (right) IHC staining of the liver metastasis resected during the initial radical surgery (September 2017), confirming the diagnosis of gallbladder adenocarcinoma and revealing HER2 protein expression. **(B)** HE (left) and HER2 (right) IHC staining of the recurrent liver metastasis resected during the second hepatectomy (March 2019), demonstrating persistent HER2 positivity. **(C)** Results of NGS detection and IHC test at several key time points. HE, hematoxylin and eosin; IHC, immunohistochemical; NGS, Next-generation sequencing.

In September 2017, he underwent hepatic resection, cholecystectomy, and lymphadenectomy. Pathology confirmed the presence of moderately differentiated gallbladder adenocarcinoma with liver invasion (pT3N1M0, according to the AJCC 8th edition, stage IIIA) (**Figure 1A**). Postoperative adjuvant therapy comprised radiotherapy in combination with concurrent S-1 chemotherapy.

**Figure 2:**
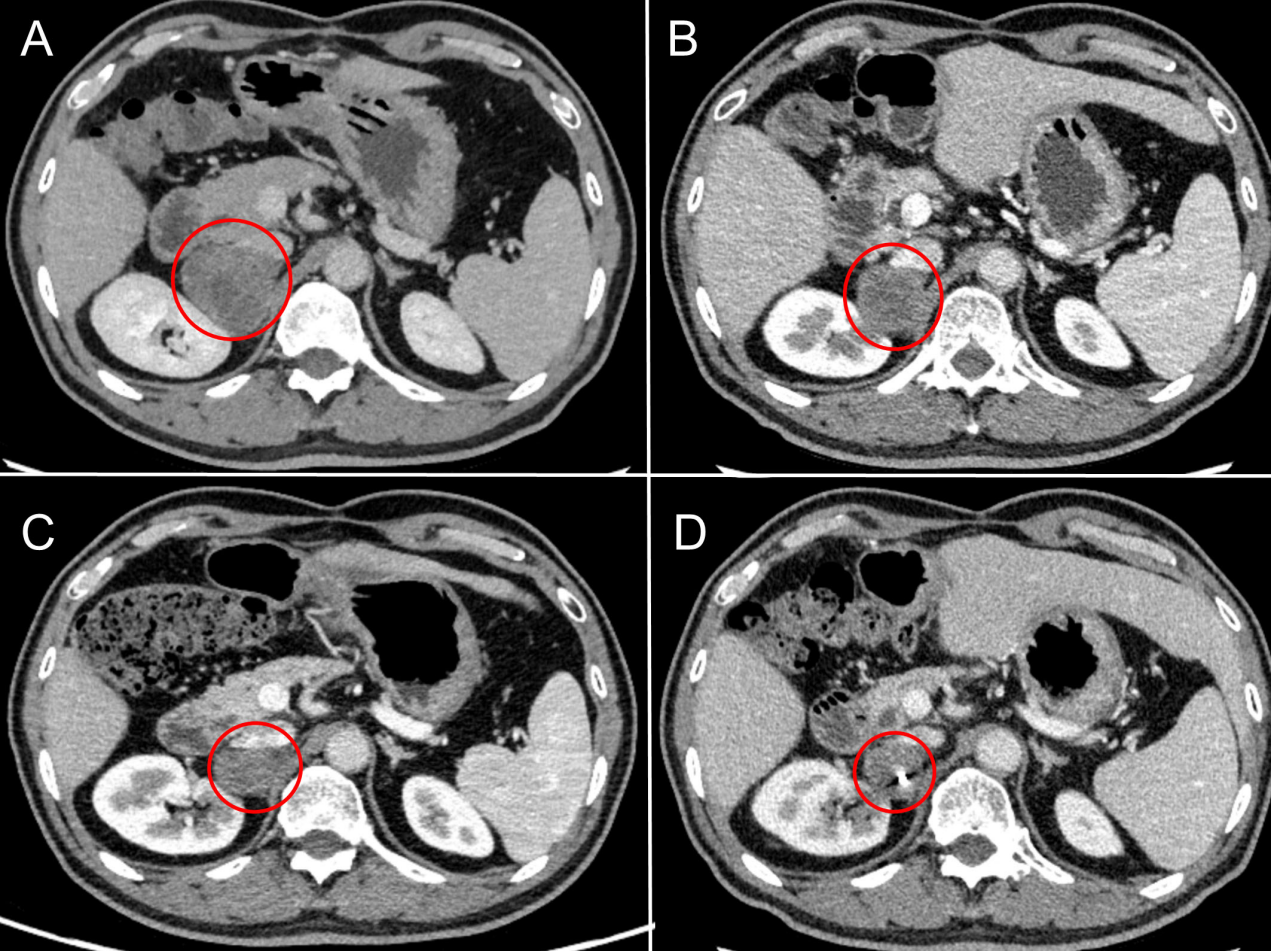
Radiographic changes in adrenal metastases under sequential local interventions. **(A)** Pre-treatment baseline (March 2020): CT scan revealing a new right adrenal metastasis. **(B)** After cryoablation (February 2021): CT image post-cryoablation shows a reduction in lesion size and density, indicating successful local tumor control. **(C)** After CyberKnife radiosurgery (February 2022): CT image demonstrates well-defined radiation-induced changes surrounding the lesion. **(D)** After odine-125 seed implantation (March 2023): CT image confirming the precise intra-tumoral placement of radioactive seeds.

**Figure 3:**
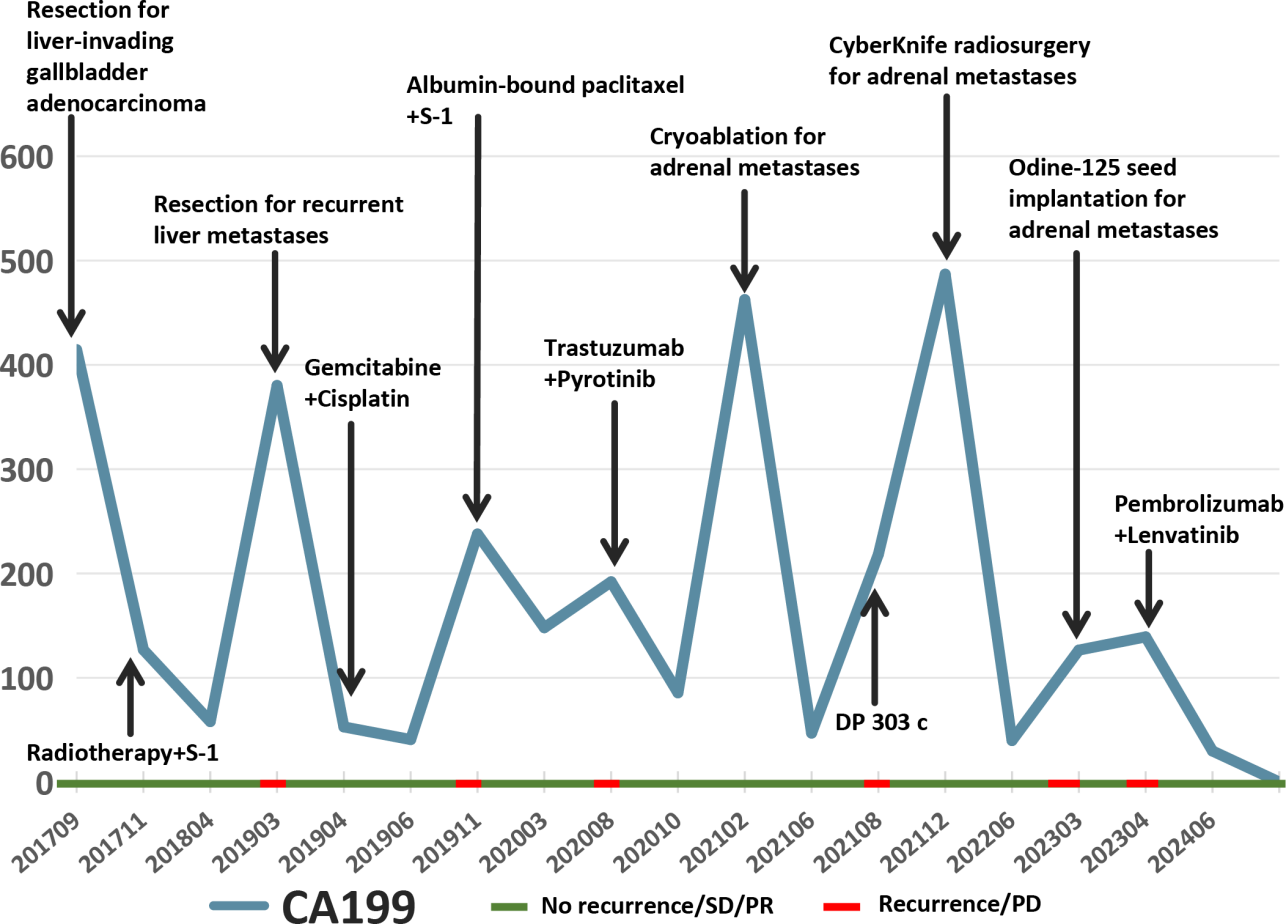
The changes in the tumor marker CA199 for this patient.

**Figure 4:**
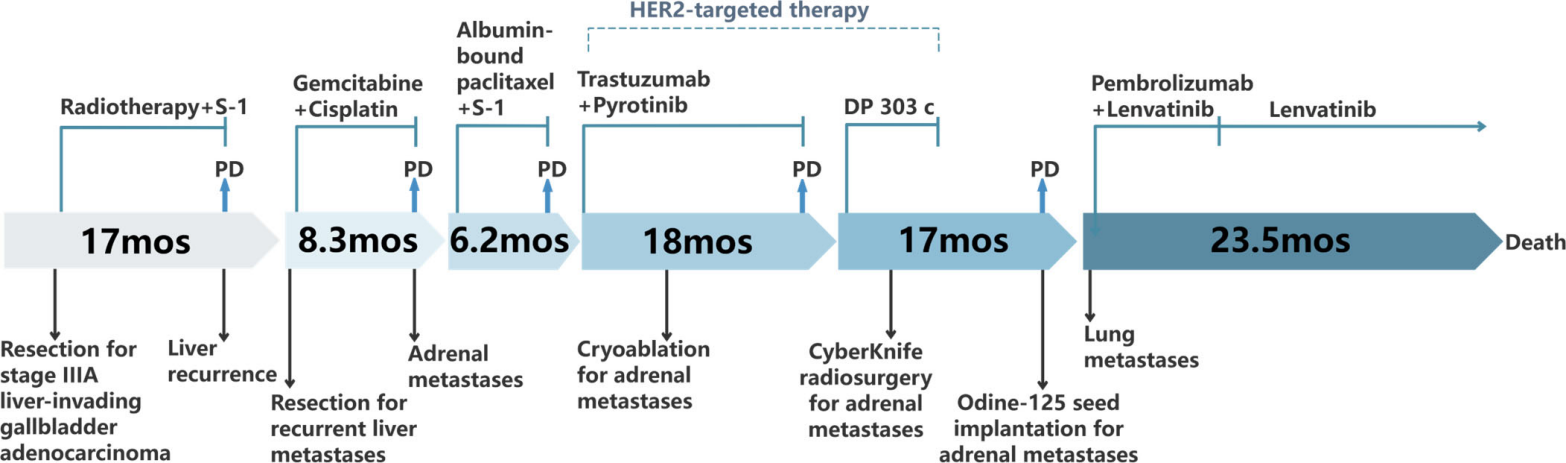
Diagram of the patient’s treatment process.

In March 2019, a second hepatectomy was performed due to hepatic recurrence, followed by four cycles of GP (gemcitabine and cisplatin) regimen chemotherapy (**Figure 1B**). Due to right adrenal metastasis, progressive disease (PD) occurred after only two cycles of AS (albumin-bound paclitaxel and S-1) regimen chemotherapy (**Figure 2A**). Next-generation sequencing (NGS) tests (Nanjing Geneseeq Technology Inc.) showed revealed ERBB2 amplification (7.4-fold) (**Figure 1C**). From May 2020 to August 2021, dual HER2-targeted therapy with trastuzumab (6 mg/kg every three weeks) and pyrotinib (400 mg daily) achieved the disease control for 18 months, during which cryoablation and pathological biopsy were conducted on the right adrenal lesion (**Figure 2B**). Due to PD in the right adrenal lesions, the patient enrolled in a phase I trial of DP303c (a HER2-targeted ADC). After two cycles, the efficacy was evaluated as stable disease (SD), but the patient withdrew after three cycles due to fatigue. The right adrenal gland was treated with CyberKnife radiosurgery in January 2022 (**Figure 2C**) and odine-125 seed implantation was performed in March 2023 due to PD in the right adrenal lesion (**Figure 2D**). The combination immunotherapy with lenvatinib (12 mg/day) plus pembrolizumab (200 mg q3w) was initiated for new lung metastases, resulting in SD. However, pembrolizumab was discontinued after two cycles due to grade 2 interstitial pneumonitis. Lenvatinib monotherapy has since maintained SD to date. The tumor marker CA199 in this patient closely mirrored the therapeutic effect, showing a significant decline following effective treatment (**Figure 3**). At the last follow-up in March 2025, the patient had achieved an OS of 90 months (**Figure 4**).

## 3 Discussion

Over the past few decades, the global mortality rate of GBC has shown a declining trend. However, due to lifestyle changes, including rising rates of overweight, the mortality rate has been on the rise in some high-income countries (13). Here, we report the case of a patient with tumor mutational burden-high (TMB-H) and HER2-positive GBC treated with HER2-targeted therapy combined with multidisciplinary management.

The next-generation sequencing (NGS) results revealed significant molecular differences in the ERBB2 gene across the three clinical subtypes of BTCs, with these variations being most prevalent in GBCs (12%-19%) (14). Patients with chemotherapy-refractory advanced BTCs harboring HER2 overexpression/amplification could receive multiple anti-HER2 treatments, including trastuzumab and pertuzumab (15), ADC trastuzumab-deruxtecan (8), the combination of trastuzumab with modified FOLFOX chemotherapy (9), the bispecific anti-HER2 antibody zanidatamab (16), and the combination of trastuzumab with tucatinib (17). In this case, the patient achieved certain efficacy with various anti-HER2 treatments and had survived for seven years.

HER2-positive BTCs show a suboptimal response to HER2-targeted therapies, which may be attributed to several resistance mechanisms. First, beyond gene amplification/overexpression, HER2 alterations in BTC include low-frequency kinase domain mutations (e.g., S310F), which drive constitutive HER2 activation and induce resistance to trastuzumab (18). Additionally, key resistance mechanisms involve reactivation of downstream signaling pathways (e.g., the PIK3CA mutation-activated PI3K/AKT pathway) (19), structural or expressiol variants of HER2 (e.g., truncated p95HER2) (20), and compensatory activation of bypass signaling such as MET or HER3 co-expression (21, 22). To overcome these limitations, dual-targeting approaches—such as trastuzumab combined with pyrotinib—emerge as promising therapeutic options (6). Pyrotinib not only effectively inhibits proliferation, migration, and invasion in trastuzumab-resistant cells, but also suppresses the PI3K/AKT and MAPK signaling pathways downstream of HER2 (23). This combination strategy significantly improves efficacy and prolongs the duration of response. Notably, PIK3CA mutations and PTEN loss are well-established negative predictive biomarkers (24, 25), while high p95HER2 expression (26) and HER3 co-expression/upregulation (27) also indicate resistance to anti-HER2 therapy, aiding in both prognostic prediction and guidance of combination strategies.

Throughout the treatment course, the patient received multi-line treatment, multidisciplinary consultations on the basis of systemic and local treatments (e.g., surgery, cryoablation, CyberKnife, and particle implantation) to further control the disease progression. Radiotherapy (RT) has served as a traditional palliative treatment for adrenal malignancies (28). However, stereotactic body radiotherapy (SBRT) offers a non-invasive ablation alternative, with evidence showing excellent local control efficacy and low toxicity rates, even for large tumors (29). This has changed oncologists’ perspectives on metastatic diseases, making it an excellent option for treating adrenal metastatic tumors (30–32). Literature indicates that monitoring adrenal hormones is essential during adrenal radiotherapy to prevent adrenal crisis, which has not been explicitly reported in the studies of SBRT (33, 34). In this case, the patient’s adrenal hormone levels were tested during the treatment process, and no abnormal changes were observed post-therapy. For metastatic diseases, although systemic treatment remains the cornerstone, its combination with local treatment may yield a synergistic effect (35). The prolonged disease remission in this patient was also attributed to the combined effect of systemic treatment and multiple local therapeutic methods. This strategy may delay the need for systemic therapy escalation and mitigate cumulative toxicity.

The advantages of the triple-therapy regimen combining gemcitabine, cisplatin, and immune checkpoint inhibitors (durvalumab or pembrolizumab) have been confirmed in the TOPAZ-1 and KEYNOTE-966 trials, marking the arrival of a new era in BTC immunotherapy. Subgroup analyses from both trials confirmed that the immune checkpoint inhibitors improved the median OS of patients with intrahepatic cholangiocarcinoma (iCCA), but there was no difference compared to extrahepatic cholangiocarcinoma and GBC (12, 36). In this case, the patient was treated with immunotherapy combined with antivascular therapy at the late line, with the recent therapeutic effect being SD. However, immunotherapy was discontinued due to immune-related adverse reactions, and the patients’ condition remained stable during subsequent continuous antivascular therapy. Various immune-mediated adverse events related to immunotherapy have also been reported (37–39). When administering immunotherapy, it is crucial to carefully assess and closely monitor related adverse reactions.

## 4 Conclusion

This case reports a HER2-positive advanced GBC patient who achieved an exceptionally long survival through HER2-targeted therapy. Based on this finding, we recommend implementing HER2 testing for all BTC patients and advocate for multi-center trials to validate HER2-directed strategies in BTCs. This case underscores the transformative potential of molecularly guided, multimodal therapy in overcoming BTC aggressiveness.

## Data Availability

The original contributions presented in the study are included in the article/supplementary material. Further inquiries can be directed to the corresponding authors.
